# Cellular and molecular mechanisms of diabetes-mediated disc degeneration

**DOI:** 10.3389/fendo.2026.1785260

**Published:** 2026-04-29

**Authors:** Hui Wang, Moran Suo, Kaizhong Wang, Shuang Chen, Xiangyan Liu, Qiwen Wang, Zhenbo Wang, Xin Chen, Zhonghai Li

**Affiliations:** 1 Department of Orthopedics, First Affiliated Hospital of Dalian Medical University, Dalian, China; 2 Key Laboratory of Molecular Mechanism for Repair and Remodeling of Orthopedic Diseases, Dalian, Liaoning, China

**Keywords:** advanced glycation end products, apoptosis, diabetes mellitus, extracellular matrix, glucose metabolism disorder, inflammation, intervertebral disc degeneration, oxidative stress

## Abstract

The aging population and lifestyle changes are contributing to a yearly increase in the incidence of spinal degenerative diseases, which significantly lead to chronic pain, neurological dysfunction, and diminished quality of life, with intervertebral disc degeneration as the fundamental pathological basis. Intervertebral disc degeneration, as the initiating factor of spinal degeneration, has always been the focus and difficulty of spine surgery. The typical intervertebral disc consists of the nucleus pulposus, annulus fibrosus, and cartilage endplate. Its nutrition primarily relies on the diffusion from the endplate, and its metabolic capacity is constrained, rendering it vulnerable to degenerative alterations caused by mechanical stress, aging, and metabolic influences. Recent studies indicate a strong correlation between diabetes mellitus and the onset and progression of intervertebral disc degeneration. As a global chronic metabolic disease, the metabolic disorders and vascular lesions caused by hyperglycemic state are closely related to spinal degenerative diseases, which makes diabetic patients face more complex spinal degeneration, and the degenerative process of intervertebral disc is accelerated and the degree of lesions is more severe. This article examines the cellular and molecular impacts of diabetic metabolic disorders on intervertebral disc degeneration, emphasizing the synergistic effects of inflammatory signaling, oxidative stress, extracellular matrix imbalance, and apoptosis. It elucidates the mechanisms by which these factors influence intervertebral disc degeneration, offering novel theoretical and practical insights for the prevention and treatment of spinal degenerative diseases, thereby furnishing patients with targeted treatment options and enhancing their quality of life.

## 1 Introduction

As the population ages and lifestyles evolve, the prevalence of spinal degeneration is rising annually, becoming a significant contributor to chronic pain, neurological impairment, and diminished quality of life (1). Intervertebral disc degeneration (IDD) constitutes the fundamental pathological underpinning of spinal degenerative illnesses, and the resultant chronic pain, neurological impairment, and necessity for surgical intervention pose significant challenges to worldwide public health (2).

IDD is regarded as the primary catalyst of spinal degeneration and has historically posed significant challenges in spinal surgical research. Normal intervertebral discs (IVD) consist of the nucleus pulposus (NP), annulus fibrosus (AF), and cartilage endplate (CEP). Their nutrition primarily relies on the diffusion from the endplate, and their metabolic capacity is constrained, rendering them vulnerable to degenerative alterations due to mechanical stress, aging, and metabolic influences (3, 4). In the initial phase of degeneration, there is a loss of water in the NP, a decrease in proteoglycans, and the development of annulus fissures, ultimately resulting in a reduction of intervertebral disc height and a loss of biomechanical function. This progression subsequently leads to various pathological changes, including intervertebral disc herniation, spinal stenosis, and lumbar spondylolisthesis (5). Recent studies indicate that diabetes mellitus (DM) is significantly associated with the onset and progression of IDD, and its metabolic disorder characteristics—such as hyperglycemia, dysregulation of glucose and lipid metabolism, and the accumulation of advanced glycation end products (AGEs)—may expedite the IDD process via direct or indirect mechanisms (6, 7). However, the precise molecular mechanism of diabetes-induced IDD remains inadequately understood, and the correlation between metabolic irregularities and intervertebral disc cell dysfunction continues to be a prominent area of research.

As a global chronic metabolic disease, the metabolic disorders and vascular lesions caused by hyperglycemic state of DM not only cause serious damage to multiple organ systems of the body, but also have been gradually found to be closely related to spinal degenerative diseases in recent years. Diabetic patients often face more complex spinal degeneration, and the progression of IDD may be further accelerated and the degree of disease may be more severe. DM can not only cause microvascular lesions in the body’s vital organs, but also affect all connective tissues, resulting in degenerative changes in bone, cartilage and IVD. Hyperglycemia and the development and buildup of advanced glycation end products are two critical characteristics of DM. These elements have been demonstrated to significantly facilitate the onset and pathological advancement of IDD, which is marked by NP cell aging, extracellular matrix (ECM) deterioration, and heightened apoptosis (8–11). In addition, a growing body of studies (12–19) suggests that DM-induced hyperglycemia and elevated AGEs can lead to IDD, which is more likely to occur in hyperglycemic environments. DM may affect the health of IVD through multiple mechanisms, and comprehending the connection between DM and IDD is crucial for advancing new prevention and treatment methodologies.

While current research has indicated a possible connection between DM and IDD from a metabolic standpoint, the majority of findings concentrate on individual pathways or molecular targets and lack comprehensive integration. This review aims to systematically clarify the cellular and molecular mechanisms of metabolic disorders in DM that impact IDD, emphasizing the synergistic effects of inflammatory signaling, oxidative stress, ECM imbalance, and apoptosis, to establish a theoretical foundation for the development of targeted metabolic therapy strategies. This paper thoroughly examines the influence of DM on IDD by synthesizing fundamental research and clinical evidence from recent years. It offers a novel theoretical framework and practical recommendations for the prevention and management of spinal degenerative disorders, aiming to furnish patients with more precise and effective treatment alternatives, alleviate disease burden, and enhance quality of life.

## 2 The basic structure and function of IVD

IVD is an important cartilage structure located between two adjacent vertebrae and is considered a shock-absorbing structure of the spine. IVD represents the largest avascular tissue in the human body, comprising three distinct parts: a gelatinous NP situated in a central region, surrounded by a lamellar AF and two CEPs positioned between the vertebral body and the AF and NP.

NP is situated between the upper and lower CEPs and presents as a milky-white translucent gel surrounded by AF.NPs are primarily constituted of chondrocytes, proteoglycan complexes, and collagen type II. Chondrocytes in NP are also known as NP cells. There are two principal types: notochord cells and mature NP cells. NP cells can produce ECM and maintain its balance, including collagen type II, proteoglycans, and water, which together provide NPs with a high degree of elasticity and resistance to compression. Therefore, IDD is associated with the degeneration or dysfunction of NP cells, which may lead to the destruction of IVD structure and functional degradation, which in turn causes Low back pain (LBP) and dyskinesia.

AF is a concentric layer structure around the periphery of the NP, each layer is tightly woven at different angles from collagen fibers, predominantly consisting of type I collagen. AF is combined with NP, and together they absorb and disperse shocks and vibrations to the spine, allowing the IVD to withstand pressure and allow the spine to flex, extend, and rotate within a certain range. Because there is no local blood supply, AF has poor repair ability once it ruptures. The injury or degeneration of the AF is the pathophysiological foundation of numerous spinal disorders, including intervertebral disc herniation and degenerative disc disease. Therefore, it is important to prevent AF injuries and treat them appropriately and promptly.

CEP is extremely important for maintaining normal IVD nutrient supply, and it acts as a mechanical barrier and solute transport between the IVD and adjacent vertebral bodies, facilitating the diffusion of small molecules and nutrient transport in IVD cells (20, 21). In addition, CEP is essential for maintaining the structure of the IVD. CEP acts as a mechanical barrier that increases the resistance to fluid flow from the IVD to the vertebral body, increases the pressure of the interstitial fluid when compressed, and prevents the IVD from protruding into the adjacent vertebral body, and this fluid pressurization helps to maintain an even distribution of stress throughout the IVD, maintaining the mechanical stability of the IVD (22). If the CEP is damaged or denatured, it may lead to the outflow of water from the NP under heavy loads, resulting in the denaturation of the NP (23).

## 3 Clinical evidence regarding DM and IDD

The predominant cause of hyperglycemia is DM, a multiorgan disorder that impacts all forms of connective tissue, including bone and cartilage. DM is widely prevalent worldwide, with 529 million patients globally in 2021, and it is projected to affect over 1.31 billion people by 2050 (24).

DM is not only manifested as blood glucose metabolism disorder, but also causes the metabolism disorder of body fat and protein, which leads to insufficient energy supply of the body, resulting in apoptosis, tissue degeneration, reduced organ function, and impacting several connective tissues, including bone and IVD. Skeletal diseases, including fractures, spinal stenosis, IDD, reduced IVD height, and diminished bone density, are prevalent among diabetic patients in clinical practice (25). DM and IDD are both one of the common diseases nowadays, and in recent years, many laboratory studies have also confirmed that abnormalities in glucose metabolism can not only interfere with the metabolism of IVD cells, causing alterations in the molecular and biochemical processes of multiple cellular pathways and apoptosis of IVD tissue cells, but also impede the transport of nutrients and metabolites from IVD tissues through the effects on CEP metabolism, causing IVD tissue degeneration (26). In addition to this, hyperglycemia can also change the composition and content of collagen in IVD tissues, causing a dysfunction of IVD tissues, which ultimately leads to degeneration of IVDs (27).

Many clinical studies have explored the link between DM and IDD. As in **Table 1**, evidence from clinical studies suggests a strong association between DM and the onset and progression of IDD. In 2010, Anekstein et al. (28) performed a cross-sectional investigation revealing a correlation between DM and lumbar spinal stenosis (LSS), proposing that DM may serve as a predisposing factor for the onset of LSS. Similarly, a cross-sectional study (30) found this conclusion. In 2016, Agius et al. (25) performed the inaugural cross-sectional study involving 100 DM patients to examine disc alterations in individuals with T2DM. Research indicates that DM may be a risk factor for IDD, as it correlates with a substantial decrease in lumbar disc height. Furthermore, lumbar disc degeneration (LDD) was found to be associated with poor control of T2DM and a longer disease duration in Chinese patients with DM, according to a retrospective single-center investigation. Furthermore, chronic DM may serve as an indicator of severe IDD (34). Jakoi et al. (31) performed a cross-sectional investigation utilizing a substantial US insurance sector database and discovered a correlation between IDD and DM. Likewise, DM has been demonstrated in a case-control study to be a risk factor for developing IDD (35). It was discovered that DM was substantially linked to IDD in the lumbar spine, particularly in the upper lumbar spine, by a long-term population-based cohort research conducted in Japan (32). According to Fabiane et al. (29), DM risk factors such age and elevated BMI exerted a more significant influence on IDD than did DM alone. A 12-year national retrospective cohort study (43) revealed that DM correlates with an elevated risk of lumbar disc herniation (LDH), and that advanced DM may signify a heightened risk of LDH. Throughout the 12-year follow-up period, the cumulative risk of LDH was markedly elevated in individuals with DM compared to those without DM. The findings indicate that patients with poorly managed DM demonstrate more pronounced IVD degradation compared to those with well-managed DM, with its effects contingent upon the length and degree of disease control. Consequently, early and rigorous glycemic management is crucial for the prevention of LDD in patients with DM.

**Table 1:** Clinical evidence regarding DM and IDD.

Year	Team	Study design	Patients	Type of DM	Outcomes
2010	Anekstein et al. (28)	A cross-sectional study	395	T1DM/T2DM	There is a correlation between diabetes DM and LSS
2016	Agius et al. (25)	A cross-sectional study	186	T2DM	Patients with T2DM are associated with a significant reduction in disc height
2016	Fabiane et al. (29)	A cross-sectional study	956	T2DM	Twins with T2DM showed higher LDD scores, but T2DM was not an independent risk factor for LDD
2016	Asadian et al. (30)	A cross-sectional, prospective study	50	T1DM/T2DM	There is a correlation between diabetes DM and LSS
2017	Jakoi et al. (31)	A retrospective cohort study	20, 000, 000	T1DM/T2DM	DM is significantly associated with an increased diagnosis of LDDD
2017	Teraguchi et al. (32)	A prospective cohort study	617	T1DM/T2DM	DM is associated with the incidence of IDD in the upper lumbar region
2017	Shakeri et al. (33)	A cross-sectional study	496	T1DM/T2DM	DM is associated with IDD
2018	Liu et al. (34)	A retrospective cohort study	772	T2DM	T2D disease lasting >10 years and poorly controlled T2DM are risk factors for IDD
2018	Steelman et al. (35)	A retrospective case control study	476, 216	T1DM/T2DM	There is a significant correlation between DM and the development of DDD
2018	Maeda et al. (36)	A cross-sectional study	968	T1DM/T2DM	DM was significantly associated with LSS in the moderate stenosis group
2020	Sudhir et al. (37)	A prospective cohort study	50	T2DM	T2DM accelerates the stress aging of human intervertebral discs
2020	Chen et al. (38)	A retrospective cohort study	118	T1DM	Glycosylated hemoglobin, venous glucose, and venous glucose fluctuation are risk factors for IDD
2020	Kakadiya et al. (39)	A Retrospective observational study	199	T1DM/T2DM	There is a positive correlation between DM and LDDD
2020	Lee et al. (40)	A prospective cohort study	2, 020	T1DM/T2DM	DM was significantly correlated with reoperation of lumbar spine surgery
2021	Park et al. (41)	A cross-sectional study	959, 360	T2DM	T2DM is significantly associated with lumbar spine disease and frequent spinal surgeries
2022	Maurer et al. (42)	A cross-sectional study	385	T1D/T2D	DM is significantly associated with disc degeneration at T7/8 and L3/4
2023	Li et al. (43)	A retrospective cohort study	19, 585, 476	T1DM/T2DM	DM is associated with an increased risk of LDH
2023	Chen et al. (44)	A prospective cohort study	259	T1DM/T2DM	Patients with DM reported worse outcomes at 1 year compared with those without DM
2023	Udby et al. (45)	A registry-based cohort study	296	T1DM/T2DM	The group with LSS and DM had an increased physical disability and worse leg pain scores at two years postoperative follow-up compared with the non-DM group
2024	Tian et al. (46)	A retrospective cohort study	262	T1DM/T2DM	Long-term uncontrolled hyperglycemia may lead to disc degeneration

IDD, Intervertebral disc degeneration; DM, Diabetes mellitus; LDD, Lumbar disc degeneration; LDDD, Lumbar disc degeneration disease; LSS, Lumbar spinal stenosis; DDD, Degeneration disc disease; LDH, Lumbar Disc Herniation; T1DM, Type 1 diabetes mellitus;T2DM, Type 2 diabetes mellitus.

A national population-based study (42) indicated that T2DM correlates with a heightened risk of concomitant lumbar spine disease and the necessity for spinal surgery. Moreover, patients with complicated DM exhibited an elevated risk for lumbar spine disorders and recurrent spinal surgeries. A retrospective analysis of 118 patients (38) revealed that individuals with T1DM displayed significant IDD, suggesting that T1DM is a risk factor for LDD. A prospective study (37) indicated that T2DM increases stress-induced senescence of IVD in humans, resulting in early IDD in patients with DM. Another prospective cohort research (44)indicated that patients with DM had poorer outcomes regarding LBP after one year compared to those without DM. DM emerged as an independent predictor of adverse patient-reported outcomes in persistent LBP. Consequently, effective glycemic management in individuals with DM may avert the onset of early IDD and inhibit the progression of the IDD cascade.

## 4 Mechanisms of IDD mediated by glucose metabolism disorder

The relationship between glucose metabolism disorders and IDD has attracted increasing attention. Research indicates that hyperglycemia can influence the advancement of IDD through multiple mechanisms. Subsequently, The link between DM and IDD will be analyzed concerning inflammatory response, ECM degradation, oxidative stress, and heightened apoptosis.

### 4.1 Inflammatory response

The high glucose (HG) condition may enhance the synthesis of inflammatory mediators, including tumor necrosis factor α (TNF-α) and interleukins (ILs), which might elevate the expression of Matrix metalloproteinases (MMPs) and A disintegrin and metalloproteinase with thrombospondin motifs (ADAMTSs), so facilitating the destruction of the ECM. DM is a chronic inflammatory disease. Clinical studies have demonstrated that DM induces IDD. As the prevalence of DM increases, DM-induced IDD is becoming a serious problem. While the mechanism via which DM promotes IDD remains unclear, inflammation is a key clinical characteristic of DM and a significant causal component in numerous disorders, including IDD. Recent investigations have shown that DM facilitates IDD via an inflammatory response, as illustrated in **Table 2**. Inflammation is a significant factor in the development of IDD (50). Degenerative IVD tissues exhibit elevated levels of numerous inflammatory cytokines, such as IL-1β, TNF-α, and IL-10 (51). Research has demonstrated increased circulation levels of proteins and some cytokines during the acute phase of DM patients (52). HG diminishes β-cell functionality and either directly or indirectly stimulates the immunological response, resulting in alterations in circulating cytokine and protein levels (52). DM individuals exhibit elevated cytokine levels, particularly TNF-α, IL-1β, IL-5, IL-6, IL-7, IL-10, and IL-18, in comparison to non-DM patients (53). In turn, these pro-inflammatory substances may result in diminished matrix gene expression and ongoing matrix disintegration by enhancing the expression of enzymes such as MMPs and ADAMTS (50).

**Table 2:** Major studies on the induction of IDD by HG through inflammation.

Year	Team	Study design	Subjects	Outcomes
2016	Park et al. (47)	*In vivo* study	Genetically Engineered Diabetes Rats	The expressions of IL-1, IL-6 and TNF-α were significantly increased in the diabetes model group
2017	Song et al. (48)	*In vitro* study	Human NP Cells	AGEs can induce inflammatory responses in NP cells through NLRP3-inflammasomes
2023	Quan et al. (49)	*In vivo* study	STZ-induced DM mouse model	DM facilitates monocyte/macrophage infiltration

IDD, Intervertebral disc degeneration; DM, Diabetes mellitus; HG, High glucose; STZ, Streptozotocin; AGEs, Advanced glycation end products; NP, Nucleus pulposus; NLRP3, NOD-like receptor thermal protein domain associated protein 3; IL, Interleukin; TNF-α, Tumor necrosis factor-α.

**Figure 1:**
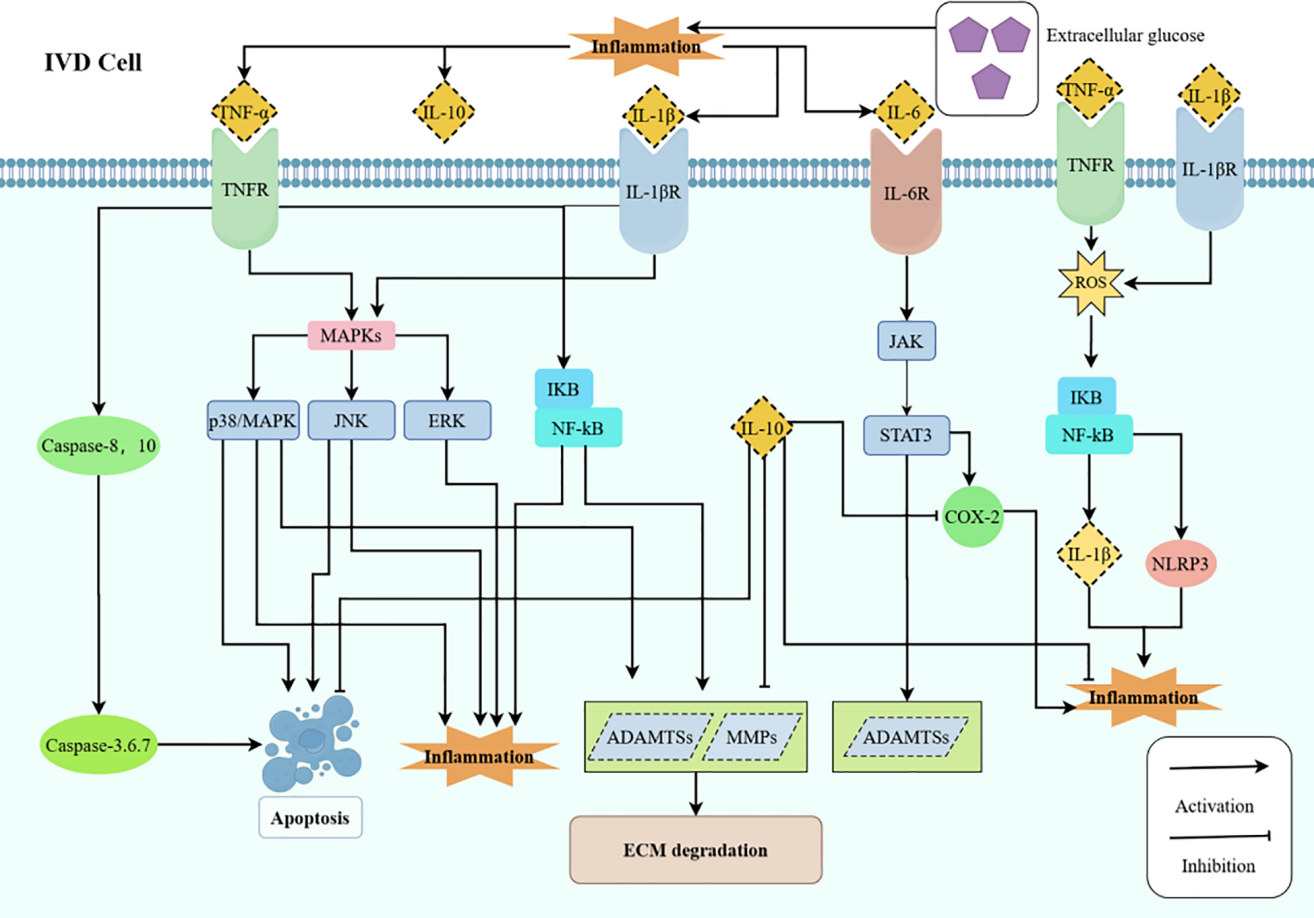
HG triggers pro-inflammatory cytokines like TNF-α, IL-1β, and IL-6 in DM, causing IVD degradation through MMPs and ADAMTSs. IL-6 promotes IDD protein production, while IL-10 reduces inflammation. IDD, Intervertebral disc degeneration; IVD, Intervertebral disk; DM, Diabetes mellitus; ROS, Reactive oxygen species; ECM, Extracellular matrix; IL, Interleukin; TNF-α, Tumor necrosis factor-α; MMP, Matrix metalloproteinases; ADAMTS, A disintegrin and metalloproteinase with thrombospondin motifs; HG, High glucose; COX-2, Cyclooxygenase-2;TNFR, Tumor Necrosis Factor Receptor; NLRP3, NOD-like receptor thermal protein domain associated protein 3.

According to certain research, IL-6 is a risk factor that has been shown to be independent of T2DM and is markedly enhanced in both T1DM and T2DM (54, 55). Plasma IL-1β levels are elevated in T1DM and T2DM as a result of IL-6-induced synthesis of High Sensitivity C-Reactive Protein (HS-CRP) (55, 56). IL-10, an anti-inflammatory cytokine secreted to mitigate inflammation, is higher in patients with T1DM and T2DM compared to healthy individuals (57, 58). Moreover, plasma TNF-α levels were markedly increased in patients with both forms of DM compared to healthy individuals. TNF-α, as a pro-inflammatory cytokine, may significantly contribute to IDD (54). Cytokines are crucial regulators of DM and IDD (59). As the degenerative process advances, increased levels of inflammatory cytokines expedite IDD by intensifying the destruction of aggrecan and collagen, while also facilitating phenotypic alterations in IVD cells (60). Furthermore, inflammatory cytokines can precipitate IVD cell death and ECM breakdown, resulting in IDD (50). DM is a long-term inflammatory illness linked to changes in several inflammatory mediators (61). Numerous cytokines are increased in individuals with DM, consequently expediting IDD. Consequently, we encapsulate the phenomena of cytokine increase in DM and succinctly examine the methods by which these cytokines expedite IDD in DM patients, as shown in **Figure 1**.

TNF-αis a cytokine that promotes inflammation. As previously stated, TNF-αlevels are frequently elevated in individuals with T1DM and T2DM. The elevated levels of TNF-α significantly contribute to the advancement of diabetic IDD by facilitating apoptosis, cellular senescence, autophagy, ECM breakdown, and inflammatory responses (62). TNF-αenhances apoptosis and elevates the levels of p53 and cleaved caspase 3 in several cell types (63). TNF-αpromotes IVD apoptosis through the activation of the JNK/ERK/MAPK and NF-κB signaling pathways (64). Furthermore, TNF-αhas been demonstrated to promote the synthesis of many pro-inflammatory cytokines, including IL-6, IL-8, IL-1β, and IL-17, by NP and AF cells, hence exacerbating the inflammatory response in inflammation-induced IDD (62). The deterioration of ECM is directly associated with the advancement of diabetic IDD, as TNF-α facilitates ECM degradation by activating various enzymes, including MMPs and ADAMTSs. These enzymes are crucial for the degradation of the ECM (62, 65, 66). Consequently, TNF-α can be regarded as a pivotal factor in the progression of diabetic IDD.

IL-1βis a pro-inflammatory cytokine. IL-1βis a crucial effector molecule in a mouse model of non-obese T1DM and serves as a significant inflammatory mediator in T2DM (67, 68). The inflammatory response is triggered by the overproduction of inflammatory cytokines, primarily IL-1β, and is strongly associated with the advancement of IDD (69). Wang et al. (70) determined that IL-1βmodulates the expression of Syndecan-4 (SDC4), facilitating aggrecan breakdown via ADAMTS-5 in the NP, and is pivotal in the etiology of degenerative disc disease. Zhao et al. (71)indicated that the stimulation of the NF-κB pathway by reactive oxygen species (ROS) facilitates NOD-like receptor thermal protein domain associated protein 3 (NLRP3) inflammasome activation and IL-1βsecretion, both of which exacerbate NP degeneration. Consequently, the elevated levels of IL-1βin DM may facilitate IDD.

Research has identified increased plasma IL-6 levels in individuals with T1DM and T2DM (54, 72). IL-6 is significantly expressed in degenerative IVDs, contributes to LBP, and possesses both pro-inflammatory and anti-inflammatory properties (73, 74). Inhibiting STAT can mitigate the detrimental effects of IL-6 in IVD, making the IL-6/JAK/STAT3 signaling pathway an attractive target for IDD treatment (75). Moreover, IL-6 enhances the production of proteins associated with IDD, such as Cyclooxygenase-2 (COX-2) and MMP13 (53). An elevated plasma IL-6/IL-10 ratio increases the incidence of IDD (73). Consequently, IL-6 assumes a multifaceted position in the advancement of diabetic IDD.

IL10 is an anti-inflammatory cytokine and a protective agent in multiple tissues, including articular cartilage and IVD tissues. Increased plasma IL-10 concentrations have been observed in individuals with T1DM and T2DM, as well as in those with degenerative IVD disease (57, 73). By inhibiting MMPs and COX-2, IL-10 therapeutically reduces the catabolic effects of pro-inflammatory cytokines (73). Behrendt et al. (76) demonstrated that IL-10 significantly decreased the synthesis of MMP-3, MMP-13, and ADAMTS-4, which are closely linked to ECM breakdown, suggesting a protective function of IL-10 on chondrocytes. Apoptosis has a significant role in the etiology of IDD (77). IL-10 improves the progression of osteoarthritis by preventing apoptosis via the reduction of activated caspase-3 levels and lowering the bax/bcl-2 ratio. Furthermore, it suppresses TNF-α-induced mitochondria-dependent apoptosis by elevating bcl-2 and down-regulating cleaved caspase-3 levels (76, 78). Thus, IL-10 is pivotal in the connection between DM and IDD.

IL-18 is a meticulously regulated inflammatory cytokine. IL-18 has been documented to be increased in T2DM (79). The role of increased IL-18 in IDD caused by DM is not well understood. IL-18 produced by pyroptotic NP cells has been demonstrated to expedite IDD by inducing degeneration of adjacent normal NP cells (80). IL-18 may influence endplate vascular endothelial cells, hence modifying the microenvironment of NP cells, AF cells, and CEP chondrocytes (53). Moreover, IL-18 has been demonstrated to damage the IVD matrix and is higher in the serum of individuals with IDD (81). Consequently, IL-18 may be a potential contributor to DM-induced IDD, but its specific molecular regulatory mechanisms and downstream signaling pathways require further elucidation.

### 4.2 Oxidative stress

**Figure 2:**
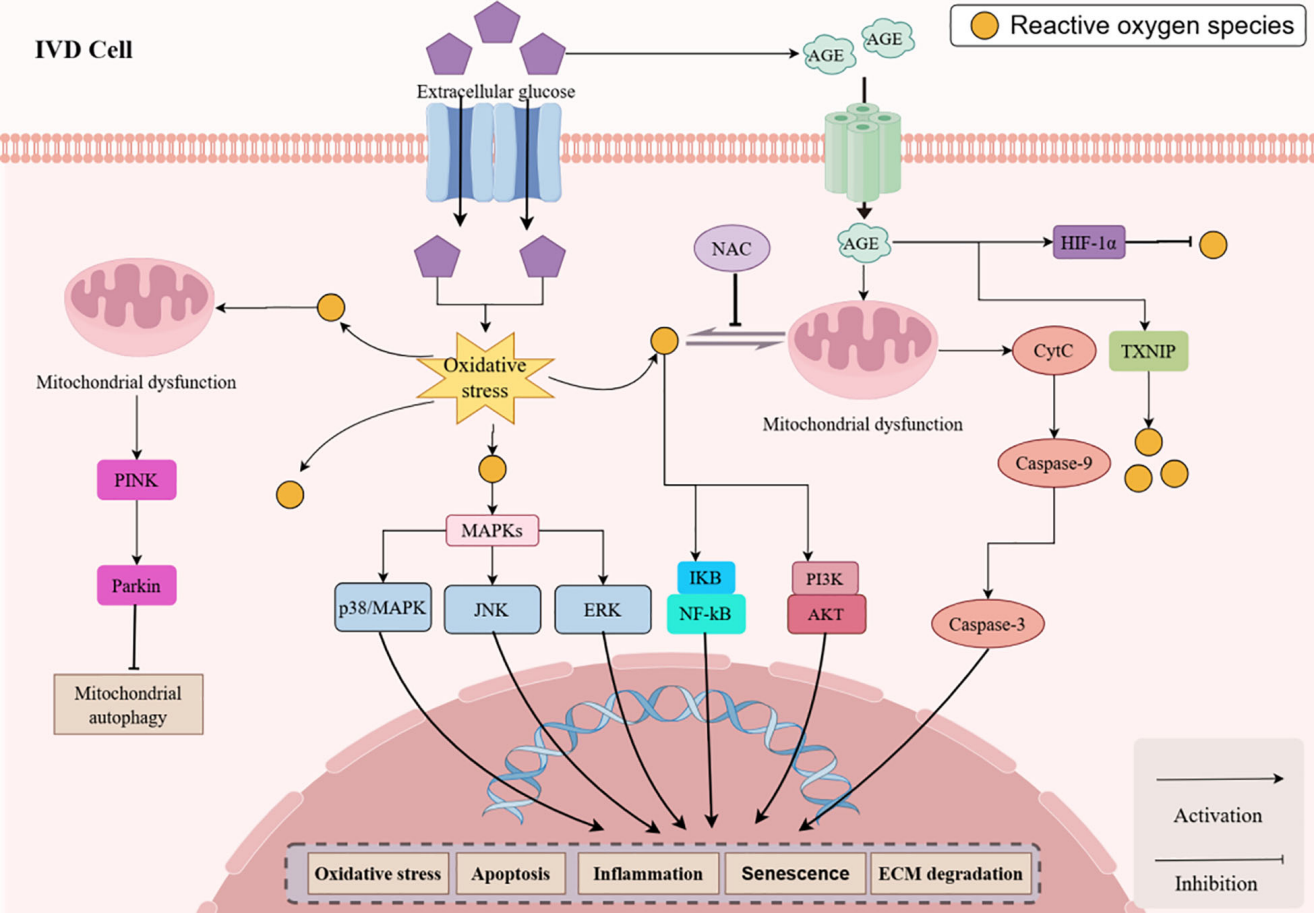
HG triggers IDD through oxidative stress and complex signaling, promoting ECM degradation and apoptosis via ROS, NF-κB, MAPKs, and PI3K/AKT pathways. IDD, Intervertebral disc degeneration; IVD, Intervertebral disk; ROS, Reactive oxygen species; HG, High glucose; ECM, Extracellular matrix; IL, Interleukin; TNF-α, Tumor necrosis factor-α; MMP, Matrix metalloproteinases; ADAMTS, A disintegrin and metalloproteinase with thrombospondin motifs; NAC, N-Acetylcysteine; AGEs, Advanced glycation end products; TXNIP, Thioredoxin-interacting protein; HIF-1α, Hypoxia-inducible factor -1α.

Oxidative stress refers to a pathological state in which excessive ROS production in organisms leads to an imbalance between oxidation and antioxidant systems, causing oxidative damage to cells or tissues. ROS are a category of free radicals linked to oxidative stress, primarily comprising superoxide anion (O-), hydroxyI radical (-OH), hydrogen peroxide (H2O2), and nitric oxide (NO). These species can be generated by mitochondria and catalase within human cells, as well as being induced by specific physicochemical conditions (82). Abundant buildup of ROS can harm biomolecules, including carbohydrates, lipids, nucleic acids, and proteins, ultimately compromising the integrity of normal cellular activity in the body (83). Oxidative stress damage induced by elevated intracellular ROS adversely affects IVD cell biology (84–86). Oxidative stress induces many phenotypes, such as IVD matrix deterioration, cellular senescence, inflammation, and apoptosis, by inflicting oxidative damage and activating detrimental signaling pathways (87–89), as shown in **Figure 2**. Recent studies have shown that DM exacerbates IDD via oxidative stress, as illustrated in **Table 3**. Oxidative stress has emerged as a prominent area of research in the treatment of IDD.

**Table 3:** Major studies on the induction of IDD by HG through oxidative stress.

Year	Team	Study design	Subjects	Outcomes
2013	Park et al. (90)	*In vitro* study	Rat NP Cells	HG increases the production of ROS in rat notochord cells
2014	Park et al. (91)	*In vitro* study	Rat AF Cells	HG-induced oxidative stress accelerates premature senescence of rat AF cells
2016	Cheng et al. (92)	*In vitro* study	Rat NP Cells	HG promotes the production of ROS in NP cells
2018	Jiang et al. (93)	*In vitro* study	Rat CEP Cells	HG induces increased ROS production in CEP cells
2019	Hu et al. (14)	*In vitro* study	Rabbit AF Cells	AGEs increased intracellular ROS production in AF cells
2021	Lo et al. (94)	*In vitro* study	Human NP Cells	The ROS of NP cells in the HG group was significantly increased
2022	Jiang et al. (19)	*In vivo* and vitro Study	Human NP Cells	HG promotes the production of ROS in human NP cells
2024	Wu et al. (6)	*In vivo* and vitro Study	Human NP Cells	HG increases ROS levels in NP cells

IDD, Intervertebral disc degeneration; ROS, Reactive oxygen species.; HG, High glucose; AF, Annulus fibrosus; NP, Nucleus pulposus; CEP, Cartilage endplate; AGEs, Advanced glycation end products.

Oxidative stress caused by ROS is acknowledged as a significant pro-apoptotic agent. Research indicates that oxidative stress can initiate the activation of apoptotic signaling pathways, including the activation of Caspase 9/3 (95). Apoptosis, as programmed cell death, plays a critical function in the elimination of damaged or unnecessary cells (96). Apoptotic signaling activates the mitochondrial malfunctioning pathway, resulting in increased intracellular ROS levels, which subsequently induces oxidative stress (97, 98). Excessive generation of ROS results in oxidative damage and cellular apoptosis (99). ROS can directly compromise mitochondrial function, therefore reducing mitochondrial membrane potential and facilitating the release of cytochrome c into the cytoplasm (100). Impaired mitochondria generate ROS, which intensify the damage and result in excessive ROS production, thereby triggering oxidative stress (101). Recent investigations indicate that the initiation and progression of IDD are intimately associated with ROS and oxidative stress. Oxidative stress increases the degradation of the ECM of the IVD and induces apoptosis, resulting in a reduction of cellular density in the IVD microenvironment, so contributing to IDD (102). Ding et al. (103)indicated that the mitochondrial apoptotic pathway was implicated in NP cell apoptosis triggered by ROS overproduction. Furthermore, cytochrome c is released from mitochondria into the cytoplasm as a consequence of the intracellular accumulation of ROS, which activates caspase and causes apoptosis (104, 105). Pioglitazone can lessen oxidative stress and mitochondrial impairment, which can prevent Nucleus Pulposus-Mesenchymal Stem Cells (NPMSCs) from undergoing apoptosis (106). Melatonin also prevents NP cells from going through apoptosis as a result of oxidative stress, as shown by He et al. (107). Moreover, liraglutide prevents HG-induced NP cell apoptosis directly by reducing oxidative stress and triggering the PI 3 K/Akt/caspase-3 signaling cascade via stimulating GLP-1 receptors (108).

Disruption of the pro-oxidant-antioxidant equilibrium within the cell results in continuous generation of ROS, which damages essential biomolecules and cells, culminating in significant inflammation (109). Across multiple signaling pathways, such as the NF-kB, MAPK, and PI3K/AKT pathways, ROS control homeostasis *in vivo* and exhibit a pro-apoptotic effect (110). These signaling pathways encompass multiple cellular processes and enable diverse physiological and pathological responses, such as cellular senescence, apoptosis, and inflammation, with the MAPK and NF-kB pathways being particularly critical. MAPK constitutes an essential signal transduction pathway in organisms. As of now, four subfamilies of MAPK have been noticed and cloned in mammalian cells: ERK, JNK/SAPK, p38/RK, and ERK 5/BMK 1. These families have a regulatory role in the initiation and progression of inflammation and can be activated by a variety of inflammatory triggers (111). Furthermore, the p53/p21 and p16/pRB pathways are the primary mechanisms that govern the aging process. These two routes are modulated by numerous variables, including oxidative damage and inflammation (112). ROS-induced mitochondrial dysfunction and oxidative stress enhance IVD cell senescence, thereby exacerbating IDD. Consequently, the key intermediary molecule ROS serves as a bridge connecting oxidative stress, apoptosis, and inflammation as major factors in the initiation and development of IDD.

ROS is advantageous to IVD cells in a physiological environment, On the other hand, an oxidative state brought on by an excessive buildup of ROS can accelerate the development of IDD (113, 114). Within the IVD milieu, oxidative stress expedites the deterioration of normal NPcells and compromises the functionality of cells that secrete collagen (115). It has been reported that in the presence of oxidative stress, IVD cells demonstrate a markedly heightened susceptibility to apoptosis, which leads to ECM breakdown and the development of IDD (116). In the setting of IDD, substantial localized ROS production indicates a relationship between IDD prevalence and increased ROS levels, offering a novel insight into IDD pathophysiology (117). Morroniside was shown to attenuate IDD, diminish NP cell senescence, and suppress the ROS-Hippo-p53 pathway by Zhou et al. (118). Guo et al. (82) demonstrated that lupinol reduced ROS-induced NP cell death in a HG environment and preserved cellular redox equilibrium. Melatonin has been demonstrated to enhance superoxide dismutase (SOD) and glutathione (GSH) activity, hence safeguarding NP cells from oxidative stress-induced mitochondrial malfunction and apoptosis. Moreover, Melatonin mitigates the compromised synthesis of collagen type II caused by hydrogen peroxide (107, 119).

Increased blood glucose levels are a significant characteristic of DM-related conditions and exert a direct or indirect influence on IDD. The HG environment in DM patients generally elevates oxidative stress-related substrates, which may represent a biochemical route associated with diabetes-related illnesses (120, 121). A wealth of evidence suggests that insufficient regulation of blood glucose levels negatively impacts physiological processes, undermines structural soundness, triggers oxidative stress and inflammatory reactions, and ultimately leads to a range of degenerative osteochondral disorders (26, 122–124). HG increases ROS levels, causes NP cells and CEP cells to undergo apoptosis, boosts ECM’s catabolic activity, and aggravates IDD (92, 93). The pathophysiology of IDD is intricately associated with ROS. Increasing data suggests that hyperglycemia may provoke oxidative stress, a primary contributor to DM-related problems (125). Hyperglycemia-induced ROS facilitate NP cell apoptosis via the mitochondrial apoptotic pathway (116). According to a study, HG concentrations in rat CEP cells triggered apoptosis in a concentration- and time-dependent way by increasing oxidative stress and impairing mitochondrial activity (111).

Studies by Won et al. (126) showed that in rat CEP chondrocytes, HG increases ROS generation, mitochondrial dysfunction, and apoptosis. Lo et al. (94)shown that DM may impede ECM formation and chondrogenesis via oxidative stress, resulting in IDD, specifically the reduction of the chondrogenic transcription factor SOX 9 and ECM proteins, including collagen type II and aggrecan. Fields et al. (127) shown in an *in vitro* investigation that T2DM diminishes GAG and water content in the IVD, elevates oxidative stress, and results in CEP sclerosis. Jiang et al. (93) demonstrated *in vitro* that HG produces apoptosis, mitochondrial damage, and elevated ROS production in CEP cells, suggesting that HG-induced oxidative stress may expedite IDD via apoptosis in CEP cells. Moreover, Park et al. discovered that HG stress induces excessive ROS generation, which hastens the senescence of rat AF cells and notochord cells via the activation of the p16-pRB pathway (91, 128).

Additionally, in immature rat notochord cells, HG-induced oxidative stress stimulates autophagy by mitochondrial impairment (90). In NP cells, oxidative stress has been found to be the primary contributor of senescence and apoptosis (92, 129, 130). ROS serve as a significant pro-apoptotic agent in the NP cells of humans, rats, and rabbits *in vitro* (131–133). In human and bovine NP cells, increased ROS production impedes ECM formation and enhances MMP expression in IVD cells, highlighting that oxidative stress plays a role in IDD progression (134–136). Previous studies have shown that the HG environment can also modulate NP cell degeneration by modulating certain signaling pathways (i.e., p38 MAPK, PI3K/Akt, and PPARγ-dependent pathways) (18, 92, 137). In addition, HG can inhibit mitophagy by modulating the ROS/PINK 1/Parkin signaling pathway (138). Mitophagy is the ability to maintain intracellular mitochondrial homeostasis and function by selectively removing damaged or malfunctioning mitochondria (139). Therefore, the HG microenvironment has been shown to promote degenerative changes in IVD cells, and inhibiting the deleterious effects induced by the HG environment is important for delaying IDD in DM patients (82, 93).

In addition, the HG microenvironment increases the production of AGEs, and the binding of AGEs to their receptors leads to localized chronic oxidative stress and induces NP cell death (140). Current research indicates that hyperglycemia markedly elevates the concentrations of AGEs and ROS in human NP cells, perhaps serving as a crucial mechanism for NP cell senescence and dysfunction (19). AGEs function as macromolecular products of non-enzymatic glycosylation processes (Maillard reactions) that enhance ROS generation and pro-inflammatory cytokine expression through interaction with their receptors (RAGE) (8, 141). The accumulation of AGEs is linked to hyperglycemia generated by DM, which leads to IDD and subsequent damaging cascade reactions. At the matrix level, DM can elevate oxidative stress expression and diminish matrix health marker expression via AGEs/RAGE-mediated interactions (127). Results from Song et al. (48) indicated that ROS were considerably increased in NP cells following treatment with AGEs. Hu et al. (14) similarly discovered that AGEs elevated intracellular ROS levels in a dose-dependent way, with ROS significantly contributing to AGEs-induced apoptosis in AF cells. They noted that the classical ROS scavenger, NAC, decreased ROS generation and mitigated AGEs-induced apoptosis. Luo et al. (142) similarly noted that AGEs treatment led to a substantial rise in the production of ROS in human NP cells and indicated the participation of the ROS pathway in the apoptosis and senescence caused by AGEs in these cells. He also pointed out that NAC somewhat reduced apoptosis and senescence brought on by AGEs therapy. Moreover, AGEs elevate mitochondrial ROS generation, prolong the mitochondrial permeability transition pore’s activation, augment intercellular ROS levels, and facilitate apoptosis in NP cells (143). Furthermore, Chen et al. (144) discovered that AGEs decreased cell survival and elevated the expression of Thioredoxin-Interacting Protein (TXNIP) in NP cells subjected to AGEs. TXNIP can expedite oxidative stress-induced cellular apoptosis by enhancing ROS production (145). Conversely, HIF-1a aids in the reduction of ROS generation in the mitochondria and the maintenance of mitochondrial function, both of which are essential for controlling cellular metabolic activity and programmed cell death (146). Xu et al. (147) did a study, a significant accumulation of AGEs was seen in the NP tissue samples of the DM group, which was adequate to induce HIF-1a degradation in NP cells. Consequently, AGEs are essential for the onset and advancement of diabetic IDD, inducing oxidative stress in IVD cells, resulting in apoptosis and senescence (113, 136, 148, 149).

### 4.3 Imbalance of the extracellular matrix

**Figure 3:**
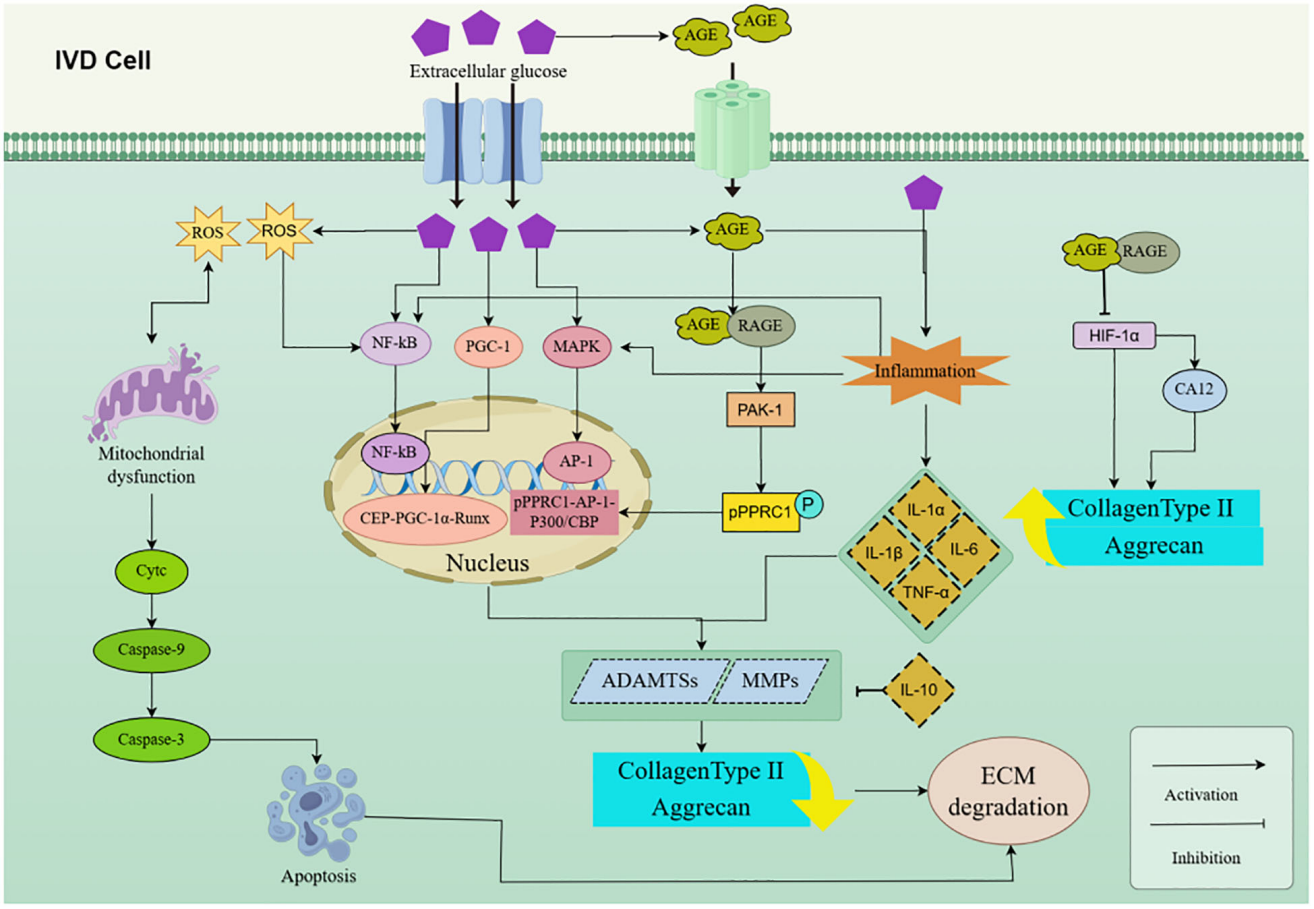
HG disrupts ECM balance, causing IDD. MMPs and ADAMTSs degrade ECM, regulated by factors like AP-1, NF-κB, Runx 2, AGEs, PAK 1, TNF-α, IL-1, HIF-1α, and ROS, contributing to IDD progression. IDD, Intervertebral disc degeneration; IVD, Intervertebral disk; HG, High glucose; ROS, Reactive oxygen species; ECM, Extracellular matrix; IL, Interleukin; TNF-α, Tumor necrosis factor-α; MMP, Matrix metalloproteinases; ADAMTS, A disintegrin and metalloproteinase with thrombospondin motifs; AGEs, Advanced glycation end products; TXNIP, Thioredoxin-interacting protein; HIF-1α, Hypoxia-inducible factor -1α; CA12.

In a healthy IVD, the production and degradation rates of the ECM are balanced due to the intricate regulation of growth factors and catabolic cytokines. The degenerative process of IDD involves ECM degradation, morphological alterations in IVD, a reduction in the number of live cells, and persistent inflammation (150). IDD is a degenerative condition characterized by ECM breakdown and apoptosis in NP cells and has been widely recognized as a contributing factor in the development and progression of LBP-related diseases (151). HG-induced alterations in IVD cells, including heightened apoptosis in NP cells and gradual degradation of the ECM, are believed to facilitate the initiation and advancement of IDD (152). Recent studies have shown that DM facilitates IDD via ECM degradation, as illustrated in **Table 4**. The mechanism by which DM promotes IDD through ECM degradation is complex, as shown in **Figure 3**. The primary constituents of the ECM of IVD are collagen type II and aggrecan, whose excessive breakdown results in the onset of IDD (115, 155). Proteoglycan and collagen loss in the ECM during IDD immediately lowers the IVD’s capacity to hydrate, which lowers the water content in the NP and ultimately causes IVD collapse and a drop in IVD height (156, 157). In diabetic IDD, HG leads to ECM degeneration in IVD tissues and AGEs significantly inhibit aggrecan expression (148, 158). An *in vivo* study (19) demonstrated that glucose concentration in the NP tissue of diabetic rats was considerably elevated compared to the control group, indicating that DM caused an elevation in glucose levels inside the NP tissue, hence affecting NP cell and tissue functionality. According to the staining results, the DM rats had fewer NP cells overall, NP cells that had undergone noticeable morphological alterations, and only minor ECM disintegration (159).

**Table 4:** Major studies on the induction of IDD by HG through ECM degradation.

Year	Team	Study design	Subjects	Outcomes
2015	Field et al. (127)	*In vitro* study	Diabetic rat coccygeal IVDs	DM compromises disc matrix homeostasis
2016	Park et al. (47)	*In vivo* study	Genetically Engineered Diabetes Rats	The expression of MMPs and TIMPs in the diabetic mouse model was significantly increased
2019	Qi et al. (153)	*In vitro* study	Human NP mesenchymal stem cells	HG treatment significantly inhibited the mRNA expression of collagen type II and aggrecans in NPMSCs
2020	Yao et al. (7)	*In vitro* study	Human NP Cells	The mRNA expression levels of collagen type II and aggrecans decreased after HNPCs were stimulated by HG
2020	Li et al. (11)	*In vivo* study	Db/db mice	Leptin receptor knockout-induced T2DM can lead to IDD by increasing MMP-3 levels
2020	Xu et al. (147)	*In vitro* study	Degenerated IVD tissue	AGEs treatment resulted in a significant increase in the expression of MMP-3, MMP-13, ADAMTS-4 and ADAMTS-5
2023	Tseng et al. (154)	*In vivo* study	HFD-induced T2DM mouse model	ADAMTS-4 and ADAMTS-5 are induced by HG
2024	Wu et al. (6)	*In vivo* and vitro study	Human NP Cells	HG promotes AGEs-induced ECM degradation in NP cells.

IDD, Intervertebral disc degeneration; IVD, Intervertebral disk.; DM, Diabetes mellitus; HG, High glucose; MMP, Matrix Metalloproteinases; NP, Nucleus pulposus; ADAMTS, A Disintegrin and Metalloproteinase with Thrombospondin motifs; ECM, Extracellular matrix; HNPC, Human Nucleus Pulposus Cell; NPMSCs, Nucleus Pulposus Mesenchymal Stem Cells; T2DM, Type 2 diabetes mellitus; TIMP, Tissue Inhibitor of Metalloproteinases; Db/db, diabetes (db) obese; HFD, High-fat diet.

MMP and ADAMTS are mainly responsible for ECM degradation (115, 155). Numerous MMPs and ADAMTSs have been found to be up-regulated in degeneration of IVDs, which are intimately linked to ECM disruption and the advancement of IDD (159, 160). MMPs, a substantial family of zinc- and calcium-dependent endopeptidases, are identified as the most essential enzymes for ECM disintegration (161). The mammalian genome has 25 MMP genes and 19 ADAMTS genes, with numerous MMP and ADAMTS members being overexpressed in degenerative IVD (115, 155). It is still mostly unclear what the underlying mechanisms are that cause MMPs and ADAMTSs to be activated during IDD. According to a number of studies, Nuclear factor-κB (NF-κB), Runt-associated transcription factor 2 (Runx 2), and Activator protein-1 (AP-1) influence the transcription of the MMPs and ADAMTSs genes (162–164). Tsang et al. (154)discovered that increased glucose levels promote the accumulation of the transcriptional activator PGC-1α, which forms a transcriptional complex with CBP and Runx 2. The CBP-PGC-1α-Runx 2 complex binds to the promoters of ADAMTS 4 and ADAMTS 5, hence promoting their expression. The overexpression of ADAMTS 4 and ADAMTS 5 expedites the disintegration of the ECM, resulting in IDD.

MMPs are the primary catabolic enzymes that facilitate the destruction of ECM components (155).Within this group of enzymes, MMP-3 and MMP-13 serve as specific and notable representatives, having demonstrated overexpression in degenerative IVD tissues and cells when compared to healthy controls. This overexpression plays a significant role in the degradation of the ECM and the ensuing progression of IDD (165). HG exposure has been shown to enhance the expression of MMP-3 and MMP-13, while reducing the levels of collagen type II and aggrecan. This finding indicates that HG promotes ECM degradation in NP cells (7). Importantly, Marein has demonstrated a significant ability to mitigate the HG-induced decline in NP cell viability, reduce apoptosis, and prevent ECM degradation through the inhibition of the ROS/NF-κB pathway (7). There is robust evidence indicating that the activation of the NF-κB signaling pathway enhances the expression of ECM components and contributes to apoptosis in NP cells, consequently facilitating the progression of IDD (166, 167).

Moreover, inflammation induces IVD cell senescence, whereas the inflammatory cytokines IL-1a, -1B, -6, -17 and TNF-a augment the catabolism of the ECM (168). Hyperglycemic stress in individuals with T2DM triggers the secretion of pro-inflammatory cytokines, including TNF-α, and IL-1, IL-6 (8, 169). The inflammatory microenvironment stimulates the production of MMPs and ADAMTS, resulting in ECM breakdown (169, 170). Recent investigations have demonstrated that TNF-a/lL-Iβ induces the production of several MMPs and ADAMTS. Shi et al. (171)proposed that IL-1β has the capacity to stimulate the production of MMP-1. Another study indicated that the stimulation of human AF cells with IL-1β resulted in a significant uptick in MMP-1 and MMP-3 levels (172). Furthermore, Zhan et al. (173)discovered that IL-1β caused a surge in the synthesis of MMP-3, MMP-9, and MMP-10, while concurrently reducing the expression of aggrecan and collagen type II. Additionally, Fang et al. (174)illustrated that IL-1β prompts the synthesis of MMP-1, -3, -13. Nonetheless, the inhibition of NF-kB markedly diminished these enzymes (166). IL-1βhas also been documented to enhance the expression of ADAMTS in the IVD (62). The co-culture of human nucleus pulposus cells (HNPC) with IL-1β activates both the NF-κB and MAPK signaling pathways, resulting in an elevation of ADAMTS-4 levels (175). Moreover, TNF-a was shown to markedly enhance the synthesis of MMP-1, -3, -13, and ADAMTS-4, -5 in isolated HNPC, resulting in the breakdown of collagen and aggrecan (175). Yang et al. (176) also reported that TNF-α stimulation resulted in a significant increase in the levels of MMP-3 and ADAMTS 5, whereas the levels of collagen type II were diminished.

NP cell senescence, apoptosis, inflammation, and oxidative stress contribute to the enhancement of ECM breakdown (177–179). The activation of autophagy can influence the activity of NP cells, therefore influencing ECM homeostasis (180). Prior research has demonstrated that inflammation can trigger ECM breakdown in the NP through the MAPK pathway (181). Research indicates that umbilical cord MSC conditioned medium (MSC-CM) can mitigate HG-induced apoptosis and ECM degradation, with the MAPK pathway being significantly involved in this mechanism (153). The expression of MMPs and tissue inhibitors of metalloproteinases (TIMPs) was markedly elevated in DM rat NP cells, whereas the presence of deteriorated ECM fractions resulted in an upsurge in MMP synthesis, subsequently generating further degraded ECM fractions (126).

Hyperglycemia and the permanent formation and AGEs are two critical features of DM that have been shown to play a role in the initiation and pathological advancement of DM-related IDD (8). The buildup of both DM and AGEs might result in many detrimental consequences on IVD, such as cellular senescence, apoptosis, and ECM destruction (182). The buildup of AGEs in the IVD resulting from hyperglycemia has been demonstrated to elevate the risk of NP cell dysfunction (148, 183). Additionally, NP cells subjected to AGE therapy have revealed diminished survival and increased ECM disintegration (48, 148). Excessive accumulation of AGEs in DM produces IDD by generating persistent abnormalities in ECM composition and vascular walls (148). In a study, 14 of the 23 examined MMPs were significantly expressed in IVD from AGEs-treated cells (184). The study’s results indicated that AGEs activated PAK 1 and caused the phosphorylation of pPPRC 1, which was safeguarded by USP 12. The stable pPPRC 1 associates with AP1-p300/CBP to create a complex that transactivates twelve MMP genes (MMP1a/1b/3/7/9/10/12/13/16/19/23/26), and the increased MMPs induce ECM degradation, resulting in IDD (184). In a polygenic obese rat model of T2DM, elevated levels of AGEs were linked to DM rather than obesity, adversely affecting IVD structure, matrix homeostasis, and biomechanical integrity. Specifically, they result in diminished ECM glycosaminoglycan (GAG) and hydration levels, augmented vertebral CEP thickness, and decreased endplate porosity (127). AGEs treatment resulted in a dose-dependent elevation in the expression levels of MMP-3, -13, and ADAMTS-4, -5, concurrently accompanied by a dose-dependent decrease in the expression of ECM component genes, including aggrecan and collagen type II (147).

Owing to insufficient angiogenesis, NP is a hypoxic tissue, resulting in the stable expression of hypoxia-inducible factor-1 a(HIF-1a) by NP cells (185, 186). HIF-1a in NP cells facilitates glycolytic metabolism and modulates ECM formation, notably increasing levels of proteoglycans and collagen type II, both of which are essential for IVD integrity (187). Furthermore, HIF-1a can enhance the production of advantageous proteins, including carbonic anhydrase 12 (CA12), a new NP marker. Reduced CA12 expression may result in diminished ECM synthesis, thereby exacerbating the course of degenerative disc disease (188). It can also diminish apoptosis in NP cells by increasing Bcl-2 expression and decreasing Bax and Caspase-3 levels (146). HIF-1a also diminishes mitochondrial ROS production and safeguards mitochondrial function, which is essential for controlling apoptosis and cellular metabolism (146). Consequently, HIF-1a is crucial for NP cells, particularly in managing hypoxic conditions and preserving cellular functioning. Xu et al. (147)noted considerable accumulation of AGEs-products in NP tissue samples from the DM group in an experimental research, and this accumulation was enough to trigger degradation of HIF-la in NP cells. In hypoxia NP cells, activation by AGEs markedly decreased HIF-1a protein levels and NP cell viability. HIF-la is a crucial regulator of NP cell viability, hence, the buildup of AGEs constitutes a risk factor for IDD (147). In addition, AGEs can lead to endplate calcification and sclerosis, microstructural alterations, ECM degradation of IVD, and an inflammatory cascade (48, 127, 148, 189, 190). DM accelerates the exacerbation of degenerative disc disease in older age by promoting the accumulation of AGEs, and the hyperglycemic state associated with DM makes IVD more susceptible to pathology by disrupting the integrity of the ECM (191). Oral administration of a combination of anti-inflammatory and anti-AGEs medications mitigates AGEs-induced ROS and inflammation in the gradually deteriorating spine of individuals with DM (192).

### 4.4 Apoptosis

**Figure 4:**
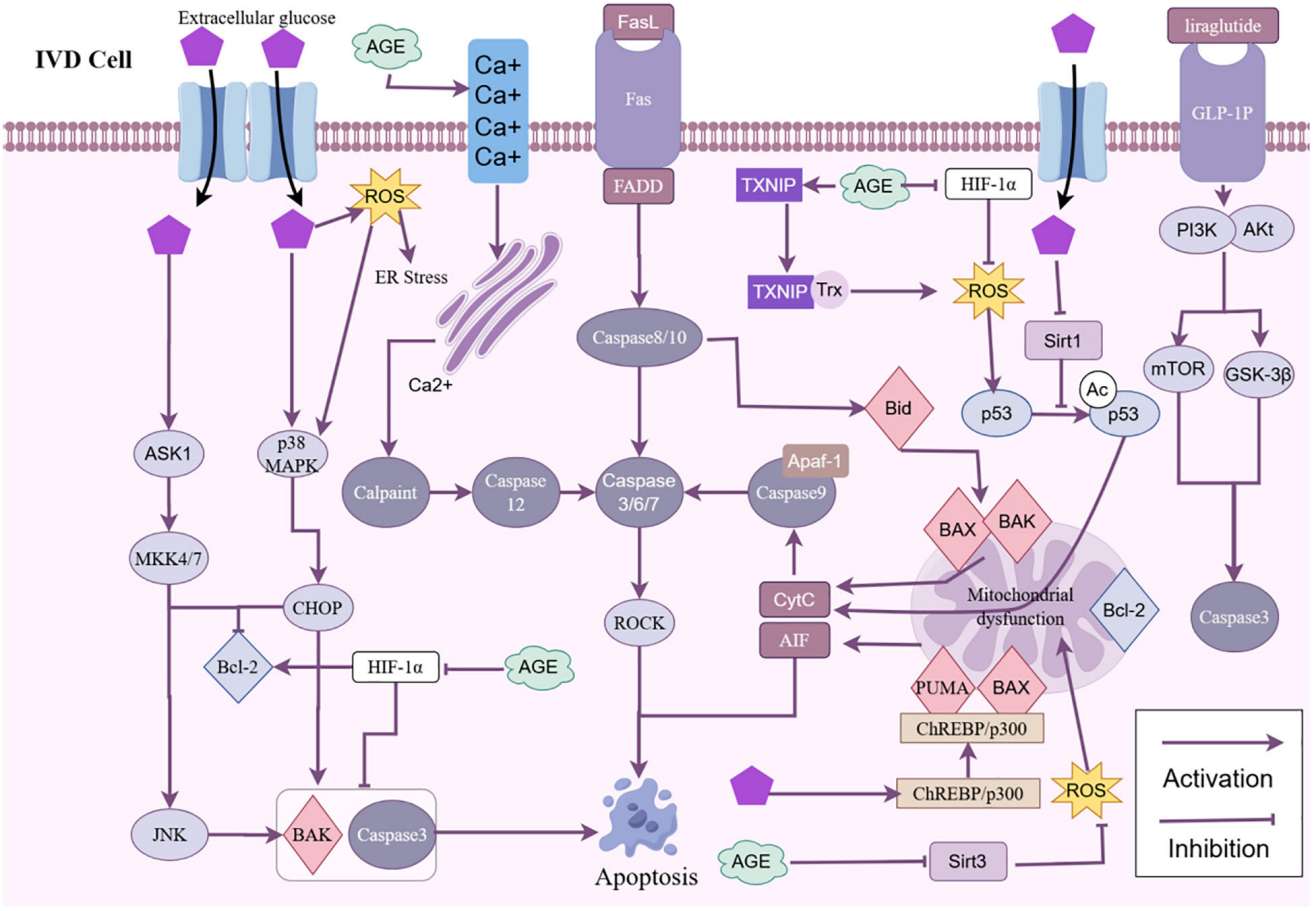
HG triggers IDD through multiple pathways, including JNK, p38 MAPK, Sirt1/P53, ChREBP/p300, Fas, ROS, AGEs, and ER stress, promoting cell apoptosis. IDD, Intervertebral disc degeneration; IVD, Intervertebral disk; ROS, Reactive oxygen species; HG, High glucose; ECM, Extracellular matrix; IL, Interleukin; TNF-α, Tumor necrosis factor-α; MMP, Matrix metalloproteinases; ADAMTS, A disintegrin and metalloproteinase with thrombospondin motifs; AGEs, Advanced glycation end products; TXNIP, Thioredoxin-interacting protein; HIF-1α, Hypoxia-inducible factor -1α; ER, Endoplasmic reticulum; AIF, Apoptosis inducing factor.

Apoptosis, or programmed cell death, is an inherent mechanism in a cell’s life cycle, but in the context of DM, this process may be expedited or excessively activated. The mechanism by which DM promotes IDD through apoptosis, as shown in **Figure 4**. In DM, apoptosis is associated with the development of multiple complications, such as the increase in apoptosis in IVD cells, which causes the ECM to deteriorate and the number of IVD cells to decline, thereby contributing to the development of IDD (193). DM is a significant factor influencing IDD, potentially altering or hastening the degradation process of IVDs by directly or indirectly impacting all facets of IDD. Hyperglycemia is a prominent characteristic of DM, which can directly influence the function of IVD cells and trigger apoptosis (9, 11). Recent studies have shown that DM facilitates IDD via apoptosis, as illustrated in **Table 5**. Consequently, HG environment-induced apoptosis of IVD cells constitutes a significant pathogenic component in DM-related spinal degeneration.

**Table 5:** Major studies on the induction of IDD by HG through apoptosis.

Year	Team	Study design	Subjects	Outcomes
2016	Cheng et al. (92)	*In vitro* study	Rat NP cells	In a HG environment, an increased rate of apoptosis in NP cells was observed
2018	Guo et al. (82)	*In vitro* study	Rabbit NP cells	In the HG environment, the apoptosis rate of NP cells was significantly increased
2018	Jiang et al. (93)	*In vitro* study	Rat CEP cells	HG induces CEP cell apoptosis
2019	Qi et al. (153)	*In vitro* study	Human NP mesenchymal stem cells	HG leads to an increase in the rate of apoptosis of NPMSCs
2019	Hu et al. (14)	*In vitro* study	Rabbit AF Cells	AGEs induced AF cell apoptosis
2019	Zhang et al. (194)	*In vivo* and vitro study	Rat NP Cells	HG enhances apoptosis of NP cells *in vivo* and *in vitro* via the sirt1/acetyl-p53 axis
2019	Shan et al. (195)	*In vitro* study	Rat AF Cells	Compared with the control group, HG culture significantly increased the apoptosis rate of AF cells
2020	Liu et al. (183)	*In vitro* study	Rat NP MSCs	The apoptosis rate of HG-NPMSCs was significantly increased
2020	Jiang et al. (196)	*In vitro* study	Rat CEP Cells	HG promotes apoptosis of rat CEP cells
2020	Yao et al. (7)	*In vitro* study	Human NP Cells	HG can induce apoptosis of HNPC
2020	Li et al. (11)	*In vivo* study	Db/db mice	Leptin receptor knockout-induced T2DM promotes apoptosis
2021	Li et al. (17)	*In vitro* study	Rat NP Cells	The apoptosis rate of NP cells cultured with HG was significantly higher than that of the control group
2021	Yao et al. (18)	*In vitro* study	Human NP Cells	The apoptosis rate of NP cells in the HG treatment group was significantly higher than that in the control group
2021	Feng et al. (15)	*In vitro* study	Human NP Cells Human AF cells	The pro-apoptotic genes Puma and BAX were significantly induced in patients with T2DM-IDD
2021	Luo et al. (142)	*In vitro* study	Rabbit NP Cells	AGEs treatment promotes apoptosis in human NP cells
2024	Chen et al. (144)	*In vitro* study	Human NP Cells	AGEs treatment promotes apoptosis in human NP cells
2024	Wu et al. (6)	*In vivo* and vitro study	Human NP Cells	HG exacerbates AGEs-induced apoptosis in NP cells

IDD, Intervertebral disc degeneration; ROS, Reactive oxygen species.; HG, High glucose; AF, Annulus fibrosus; NP, Nucleus pulposus; CEP, Cartilage endplate; AGEs, Advanced glycation end products; NPMSCs, Nucleus pulposus mesenchymal stem cells; T2DM, Type 2 diabetes mellitus; IDD, Intervertebral disc degeneration; Db/db, diabetes (db) obese; HNPC, Human Nucleus Pulposus Cell.

Apoptotic signaling pathways are categorized into exogenous routes (death receptor pathways) and endogenous pathways (mitochondrial pathways), where exogenous pathways are activated by death ligands and receptors, and endogenous pathways are stimulated by cellular stress (197). Apoptosis in healthy organisms is rigorously regulated; nonetheless, hyperglycemia induces apoptosis by activating exogenous pathways mediated by dead cell receptors and endogenous pathways that mobilize mitochondria and endoplasmic reticulum (ER) pathways (26). In the DM rat model, Jiang et al. (198)discovered that NP cell apoptosis was elevated in DM rats, activating both exogenous and endogenous mechanisms to intensify apoptosis. Won et al. (126) shown that hyperglycemia in diabetic rats results in heightened apoptosis and premature apoptosis of IVD notochordal cells, which subsequently change into a fibrocartilaginous matrix as the IVD advances.

According to Yao et al. (18), HG treatment markedly up-regulated the expression of caspase 3 and Bax, while down-regulating the expression of Bcl 2, an anti-apoptotic molecule. Furthermore, the results suggest that liraglutide inhibits HG-induced apoptosis in NP cells via triggering the PI3K/Akt/mTOR/Caspase-3 and PI3K/Akt/GSK3 β/Caspase-3 signaling pathways. Similar findings were made by Li et al. (17)in their investigation, which showed that the HG group had significantly higher Bax, autophagy-related molecule mRNA or protein expression, a significantly higher apoptosis rate, and significantly lower Bcl-2 mRNA expression when compared to the control group. The results also demonstrated that melatonin supplementation in the HG group prevented NP cell apoptosis via reducing HG-induced hyperautophagy in NP cells. Furthermore, by inducing autophagy, metformin can shield NP cells from senescence and apoptosis (129). Zhang et al.’s research (194) demonstrated that DM could accelerate the course of IDD by speeding up NP cell apoptosis and senescence, while Butein might ameliorate diabetic IDD by shielding NP cells from apoptosis and senescence via the Sirt 1/P53 axis. The NAD-dependent deacetylase Sirt1 has been shown to be inhibited by hyperglycemia in NP cells, both in terms of expression and activity. Prior research has also shown that hyperglycemia reduces Sirt1 expression, resulting in elevated p53 acetylation (199). p53 serves as a prevalent regulator of senescence and apoptosis (200). Feng et al. (15) demonstrated that HG can expedite the progression of IDD in rat models, where in HG stimulates the ChREBP/p300 transcriptional complex and interacts with the Puma and BAX promoters, contributing to the pathogenesis of apoptosis and IDD. Puma and BAX were considerably upregulated in patients with T2DM-IDD, with their expression levels stimulated by glucose. The aggregation of Puma and BAX may induce mitochondrial malfunction and activate caspases, resulting in apoptosis. Moreover, hyperglycemia elevates glucose levels within the tissue microenvironment. Recent investigations indicate that elevated glucose levels markedly inhibit the growth of NPMSCs while enhancing their senescence and apoptosis (183).

In a rat model of DM caused by streptozotocin (STZ), DM has been shown to intensify apoptosis by activating the apoptosis signaling system, leading to significant degradation of the ECM and dysregulation of proteases, thereby hastening the progression of IDD (198). In an experimental study using a STZ-induced DM rat model, Kong et al. (201)observed that the STZ group had significantly higher expression of caspase -3, -8, -9, and Fas. It was also discovered that in a rat model of DM, insulin therapy reduced excessive apoptosis and matrix breakdown of IVD cells. Fas is a recognized apoptotic receptor, and Fas expression was reported by Park et al. (202, 203)in both human and animal IVD cells, including NP and AF cells. In a genetically modified rat model of DM, hyperglycemia results in a decrease in the ECM and apoptosis of IVDs, linked to the Fas-mediated apoptotic signaling pathway (47). Fas is a cell-surface death receptor that, when binding with its ligand FasL, causes NP cells to undergo excessive apoptosis by means of the type I (caspase-8) and type II (caspase-9) pathways mediated by Fas (201).

Moreover, hyperglycemia may intensify apoptosis and matrix degradation by influencing additional signaling pathways, including the p38 MAPK pathway. In *in vitro* culture experiments of human NP, QI et al. (153)demonstrated that HG induces increased phosphorylation of p38 MAPK in NPMSCs and significantly elevates apoptosis. The use of p38 MAPK inhibitors effectively mitigated the phosphorylation induced by HG, offering potential therapeutic strategies for IDD in diabetic patients. IncRNA MALAT1 significantly facilitates apoptosis in rat CEP cells through the activation of the p38 MAPK signaling pathway (196). This indicates that MALAT1 may serve as a therapeutic target for DM-related IDD. p38 MAPK is a crucial signaling molecule for cellular responses to inflammation. Under diabetic mellitus conditions, hyperglycemia may elevate the death rate of IVD cells by activating the p38 MAPK pathway, resulting in IDD.

Oxidative stress is a significant contributor to IVD cell death and senescence. Oxidative stress has been demonstrated to initiate apoptosis-related signaling pathways, including the activation of caspase 9 and 3 (95). Hyperglycemia has been demonstrated to induce oxidative stress, activating the p38 MAPK signaling pathway, which subsequently facilitates apoptosis and stromal descent in IVD cells (92). Furthermore, HG-induced ROS can facilitate apoptosis in NP cells via the mitochondrial apoptosis route (116). Jiang et al. (93) demonstrated that HG-induced excessive ROS production can induce apoptosis in CEP cells via mitochondrial impairment, whereas a-lipoic acid (ALA) can mitigate mitochondrial damage and apoptosis in CEP cells triggered by HG stimulation, indicating a potential correlation between HG-induced oxidative stress and IDD, Guo et al. (82) demonstrated through *in vitro* tests that excessive ROS generation is a primary component contributing to NP cell death during hyperglycemia. Guo et al. (82)further established that the excessive generation of ROS is a primary contributor to the apoptosis of NP cells in hyperglycemic conditions. Lupeol was discovered to impede HG-induced apoptosis of NP cells by augmenting the mitochondrial antioxidant stress response. Wu et al. (6) noted that AGEs prompted degeneration of NP cells by elevating ROS. Hyperglycemia exacerbated ROS levels by impairing AGEs-induced protective mitophagy, consequently increasing AGEs-induced apoptosis. Furthermore, exogenous vascular endothelial growth factor A (VEGFA) mitigated AGEs-induced apoptosis in NP cells, potentially offering a therapeutic approach for DM-associated IVD degeneration. Simultaneously, Yang et al. (204)exhibited through *in vitro* research that the connection between 17β-estradiol and estrogen receptorβ mitigates apoptosis and enhances matrix synthesis in NP cells by reducing oxidative damage under HG settings. Furthermore, ER stress can impair several cellular homeostatic functions, resulting in apoptosis and degeneration of the IVD.

Two key features of DM that greatly contribute to the start and catastrophic progression of DM-related IDD are hyperglycemia and the irreparable production and accumulation of AGEs (8). The accumulation of AGEs in DM might result in oxidative stress, ER stress, and autophagy, trigger apoptosis, and decrease the quantity of IVD cells and NP cells, thus leading to IDD. According to Luo et al. (142), treating human NP cells with AGEs caused them to die and get old, demonstrating that FAM134B, a mammalian ER-phagocytic receptor, mediates ER-phagocytosis activated by the ROS pathway in response to AGEs. Significantly, they discovered that the over-expression of FAM134B reduced the accumulation of ROS and apoptosis in AGEs-treated NP cells. The production of proteins, maturation, and quality assurance are all under the ER’s purview. The accumulation of unfolded or misfolded proteins, referred to as ER stress, is caused by genetic and environmental stresses that affect protein folding in the ER. However, persistent ER stress can cause cells to destroy themselves (205). A vital physiological regulator, Ca2+ is involved in many cellular functions, including metabolism, apoptosis, protein folding and modification, and gene transcription (206). Regulated Ca2+ homeostasis is essential for proper ER function, while its dysregulation results in the buildup of protein fold errors and triggers the ER stress-induced apoptotic pathway (207). AGEs result in ER stress by prolonging the increase of cytoplasmic Ca2+ in NP cells and reducing Ca2+ in the ER lumen in a manner that is dependent on both concentration and time (208). In NP cells treated with AGEs, Luo et al. (209) found that the ER stress response was enhanced in a time and concentration-dependent manner. Thus, AGEs-induced ER stress and the subsequent apoptosis of NP cells are greatly influenced by disturbed Ca2+ homeostasis. Furthermore, Xu et al. (147)noted substantial accumulation of AGEs in DM-NP tissue samples during an experimental research, adequate to trigger HIF-1a breakdown in NP cells. AGEs buildup is a risk factor for IDD, as HIF-1αregulates NP cell survival by boosting Bcl-2 expression and lowering Bax, and Caspase-3, -9 expression (147).

Furthermore, AGEs may block Sirt3 action and induce apoptosis and IDD in NP cells. AGEs disturb mitochondrial redox equilibrium by compromising the SIRT3-mitochondrial antioxidant system. This elevates intercellular ROS levels and facilitates apoptosis in NP cells (143). TXNIP functions as an inhibitor of the thioredoxin system. TXNIP has been shown to trigger apoptosis in NP cells under conditions of oxidative stress (210). AGEs impede glycolysis by enhancing TXNIP expression, which is implicated in AGEs-induced death and decreased viability of NP cells (144). Consequently, AGEs are a crucial element in the mechanism of DM-induced IDD.

### 4.5 Other

DM can initiate and accelerate IDD by mediating pyroptosis in NP cells. Hyperglycemia and metabolic dysregulation induce inflammatory responses and activate the HMGB1-TLR4 pathway, concurrently triggering oxidative stress in NP cells and promoting excessive ROS production. These dual mechanisms jointly activate the NLRP3 inflammasome, subsequently activating caspase-3 through the classical pyroptotic pathway, cleaving GSDMD to directly induce pyroptosis in NP cells (211). Concurrently, DM downregulates anti-pyroptotic factors such as BMP7, diminishing their inhibitory effects on NLRP3 and pyroptosis while disrupting ECM metabolic equilibrium. Moreover, a HG environment may upregulate the expression of pyroptosis-related molecules such as NLRP3 and ASC, exacerbating endplate sclerosis and vertebral osteoporosis, thereby further promoting matrix degradation and the progression of idiopathic disc degeneration. Euphronol, however, can delay this process by inhibiting pyroptosis-related pathways (212).

Autophagy dysfunction is a critical factor in diabetes-mediated disc degeneration, primarily driven by a HG environment and the accumulation of AGEs. HG induces oxidative stress and excessive ROS production, impairing mitochondrial function. On one hand, this activates autophagy; on the other, it inhibits mitochondrial autophagy via the ROS/PINK1/Parkin pathway, leading to the accumulation of damaged mitochondria and exacerbating oxidative damage (90, 138). Concurrently, HG levels may trigger excessive autophagy in NP cells via PPARγ-dependent pathways, leading to cellular dysfunction (137). Upon binding to the receptor RAGE, AGEs further exacerbate oxidative stress and ER stress, disrupting the expression of autophagy-related proteins and synergizing with HG to intensify intervertebral disc cell damage (209, 213, 214). Inflammatory mediators such as TNF-α can simultaneously regulate autophagy and apoptosis, jointly promoting ECM degradation (62). Metformin mitigates cellular damage by inducing protective autophagy, while melatonin suppresses pathological excessive autophagy, suggesting that restoring autophagy balance represents a potential therapeutic approach for improving diabetes-related IDD (17, 129).

DM accelerates the progression of IDD by inducing premature senescence of intervertebral disc cells, with HG levels and AGEs serving as key pathogenic factors. A HG environment elevates ROS levels, induces oxidative stress and mitochondrial damage, activates aging pathways such as p53/p21 and p16/pRB, inhibits Sirt1 activity, and enhances p53 acetylation, leading to stress-induced premature aging in cells such as NP cells and AF cells, manifested as reduced proliferation and matrix synthesis capacity (91, 92, 128, 194). AGEs exacerbate oxidative stress and endoplasmic reticulum stress via RAGE, activating inflammatory pathways and synergizing with HG to accelerate cellular aging (19, 142). Diabetes-associated inflammatory mediators such as TNF-α and IL-1β further amplify aging effects through NF-κB and MAPK pathways, ultimately leading to reduced synthesis of type II collagen and proteoglycans and disruption of ECM homeostasis (62, 71). In terms of interventions, metformin and melatonin may mitigate cellular aging by regulating autophagy, providing antioxidant effects, and activating Sirt1 pathways. Butein, meanwhile, may exert anti-apoptotic and anti-aging effects via the Sirt1/p53 axis, offering potential strategies for delaying IDD (17, 129, 194).

## 5 Pharmacological treatment of IDD mediated by DM

Metformin, as the first-line treatment of choice for T2DM, demonstrates significant therapeutic potential in the field of IDD. This medication induces protective autophagy by activating the AMPK signaling pathway, effectively inhibiting apoptosis and senescence in NP cells. Concurrently, it positively regulates ECM metabolism by promoting the synthesis of type II collagen and aggregated proteoglycans while suppressing the activity of matrix-degrading enzymes such as MMP3, thereby maintaining the structural integrity of the intervertebral disc matrix (129). Moreover, metformin alleviates local inflammatory responses within the intervertebral disc by inhibiting the NF-κB signaling pathway and blocking HMGB1 release. It further enhances the secretion of mesenchymal stem cell exosomes and inactivates the cGAS/STING pathway, thereby ameliorating cellular senescence and inflammatory stress states (168). Although no clinical trials for IDD have yet been conducted, given the pathological association between metformin and IDD, coupled with the drug’s established clinical safety profile and robust research foundation, metformin holds promise as an adjunctive therapy for IDD. It is particularly suitable for IDD patients with concomitant T2DM.

Beyond metformin, other classes of hypoglycemic agents also demonstrate potential value in IDD interventions. Sitagliptin, a dipeptidyl peptidase-4 (DPP4) inhibitor, has been confirmed through Mendelian randomization studies to target a causal risk factor for disc degeneration, with DPP4 exhibiting high expression in degenerative NP tissue. Sitagliptin can delay the progression of disc degeneration in rats by inhibiting macrophage infiltration and M1-type polarization, blocking the NF-κB/NLRP3/IL-1β inflammatory axis, and thereby mitigating inflammatory damage to NP cells and ECM degradation (207). Liraglutide, as a GLP-1 receptor agonist, can inhibit hyperglycemic environment-induced NP cell apoptosis and oxidative stress by activating the PI3K/Akt/caspase-3 pathway (108). Pioglitazone, as a PPAR-γ agonist, can reverse the downregulation of PPAR-γ in degenerative disc tissue. By inhibiting the NF-κB pathway, it reduces the release of pro-inflammatory mediators such as IL-17 and TNF-α, along with matrix metalloproteinases, thereby alleviating disc inflammation and structural degeneration (215).

Beyond hypoglycemic agents, various small-molecule compounds and biological agents may also intervene in the IDD process through antioxidant, anti-inflammatory, and matrix homeostasis-maintaining mechanisms. 17β-estradiol alleviates oxidative damage in NP cells under hyperglycemic conditions by binding to the estrogen receptor β (204). Marein can eliminate high-glucose-induced ROS accumulation, inhibit NF-κB pathway activation, and mitigate oxidative damage in NP cells (7). Lupinol enhances mitochondrial antioxidant capacity by upregulating Bcl-2, downregulating Bax and apoptosis-related protease expression, thereby suppressing high-glucose-induced NP cell apoptosis (82). Melatonin reduces ROS levels, enhances superoxide dismutase and glutathione activity, maintains mitochondrial function, thereby decreasing NP cell apoptosis and protecting the extracellular matrix (107). Regarding the accumulation of AGEs, the combination of the anti-inflammatory agent polysaccharide polysulfate and the anti-glycation agent pyridoxamine reduces the accumulation of advanced glycation end products in spinal tissues of diabetic mice. This synergistically inhibits inflammatory responses and catabolic processes, thereby preserving the structural integrity of IVD (192). Furthermore, conditioned medium derived from umbilical cord blood mesenchymal stem cells can restore the matrix synthesis capacity of NP mesenchymal stem cells under hyperglycemic conditions by modulating the p38 MAPK pathway, offering a novel therapeutic strategy for regenerative treatment of disc degeneration (153).

Currently, exploring novel pharmacological treatment strategies for IDD, particularly intervention protocols for IDD patients with concomitant DM, has become a significant research focus in this field. The diverse mechanisms of action demonstrated by various drugs suggest that targeted interventions hold considerable potential in slowing the pathological progression of IDD. Nevertheless, it is noteworthy that elucidating the interactions between novel drugs and conventional hypoglycemic agents, along with their impact on glucose regulation, is crucial for ensuring clinical safety. Given that both IDD and DM are chronic progressive conditions, conducting long-term longitudinal studies to systematically evaluate the long-term safety and therapeutic efficacy of relevant drugs in IDD patients holds significant clinical importance.

## 6 Conclusion and prospects

IDD is a multifactorial disease, and the effect of hyperglycemia and AGEs on IDD is an important area in DM research. Glucose metabolism disorder, especially hyperglycemic states, have a significant effect on IDD. Hyperglycemia can directly harm IVD cells, leading to cellular senescence and apoptosis, which in turn promotes IDD. In addition, hyperglycemia leads to the accumulation of AGEs, which can exert deleterious effects on IVD cells, exacerbate ECM degradation and apoptosis, and thus promote IDD.

There exists a close association between DM and IDD. Reliable laboratory data have confirmed that glucose metabolism disorders disrupt the homeostasis of IVD cells through multiple molecular and biochemical pathways, ultimately leading to cell apoptosis and tissue degeneration. Nevertheless, significant research gaps remain. Firstly, the molecular mechanisms underlying the interaction between impaired glucose metabolism and the intervertebral disc (IVD) microenvironment remain incompletely elucidated, particularly concerning the specific roles of inflammation, matrix catabolism, oxidative stress, and apoptosis in this process. Secondly, the limited number of clinical studies, insufficient sample sizes, inconsistent observational findings, and lack of high-quality controlled trials impede the translation of these research discoveries into clinical practice.

To address these gaps, future research should prioritize the following directions: (1) Conducting multicenter, large-sample clinical studies to establish standardized diagnostic and treatment protocols for IDD with DM; (2) Investigate targeted interventions across multiple core pathways and develop novel delivery methods for candidate drugs demonstrating beneficial effects in mitigating high-glucose-induced IDD, such as liraglutide, resveratrol, cycloastigenol, astragaloside IV, and melatonin (16, 18, 216);(3) Explore the therapeutic potential of mesenchymal stem cells and exosomes in repairing DM-mediated IDD.

In conclusion, robust laboratory data and insufficient clinical trials indicate that DM is a contributing factor to IDD. The effects of hyperglycemia and AGEs on IVD apoptosis are multifaceted, involving multiple pathways such as inflammation, oxidative stress, matrix catabolism, autophagy, senescence and apoptosis. Future studies need to further reveal the specific mechanisms of these processes and develop more effective therapeutic strategies. Only a full understanding of how they interact with each other and the changes that occur during degeneration will be more conducive to grasping the pathomechanism of IDD and provide a more accurate theoretical basis for further clinical treatment and prevention.
